# Evaluation of the anaphylactoid potential of *Andrographis paniculata* extracts using the popliteal lymph node assay and P815 cell degranulation *in vitro*

**DOI:** 10.1186/s12967-015-0478-0

**Published:** 2015-04-14

**Authors:** Xuguang Hu, Ya Wen, Shasha Liu, Jiabo Luo, Xiaomei Tan, Zhiheng Li, Xinhua Lu, Xiaoying Long

**Affiliations:** College of Traditional Chinese Medicine, Guangdong Pharmaceutical University, Guangzhou, 510006 China; Zhongshuai Pharmaceutical Sci&Tech Incorporated Co., LTD, Zhengzhou, 450000 China; Key Laboratory of New Traditional Chinese Drugs, School of Traditional Chinese Medicine, Southern Medical University, Guangzhou, 510515 China; Department of Pharmacy, Affiliated Hospital of Armed Police Logistics College, Tianjin, 300000 China

**Keywords:** Andrographis extracts, D-PLNA, Anaphylactoid reaction, Mast cell degranulation

## Abstract

**Background:**

The anaphylactoid reactions induced by andrographis injection have repeatedly been reported. The aim of our study was to evaluate the immuno-sensitizing potential of extracts from *Andrographis paniculata* Nees and to screen for the constituent that is responsible for inducing the anaphylactoid reaction.

**Methods:**

In the direct popliteal lymph node assay (D-PLNA), female BALB/c mice were randomly divided into several groups with ten mice per group according to the experiment design, the right hind footpads of mice received a single subcutaneous injection of *Andrographis paniculata* (50 μl), and the left hind footpads received the same volume of vehicle. Seven days later, the mice were sacrificed by cervical dislocation, and the popliteal lymph nodes from both the left and right sides were removed. The weight (WI) and cellularity indices (CI) of the popliteal lymph nodes (PLNs) were then calculated, and the pathological changes of the PLNs were measured. In addition, P815 mast cells were collected for the *in vitro* cell degranulation experiment. The level of histamine, the percentage of cell degranulation and the ratio of ammonia glycosidase released were measured to further evaluate the potential allergenicity.

**Results:**

Alcohol extract (AEE), ethyl acetate extract (EAE) and n-butanol extract (NBE) significantly increased the weight (WI > 2) and cell number (CI > 5) of PLNs (P < 0.05, P < 0.01). Additionally, all the three monomers of andrographis, namely NAD, AND, and DDA, significantly increased the weight (WI > 2) and cell number (CI > 5) of the PLNs (P < 0.05, P < 0.01). In the cell model, all of the different extract fractions (AEE, EAE and NBE) and the three monomers of andrographis markedly elevated the level of histamine, the percentage of cell degranulation and the ratio of ammonia glycosidase released.

**Conclusion:**

The diterpene lactone compounds of *Andrographis paniculata* Nees (total lactones of andrographolide) may have a potential sensitizing capacity in andrographis injection.

## Background

**Figure 1:**
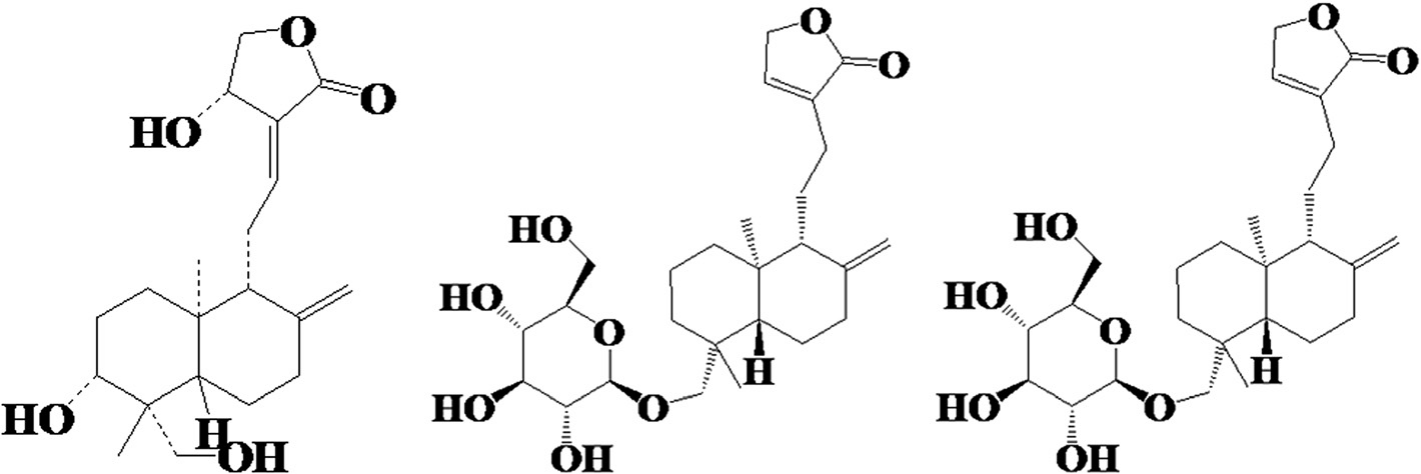
Chemical structures of the major active ingredients from andrographis: diterpene lactone compounds.

*Andrographis paniculata* (Burm. F.) Nees, an herbal plant of the Acanthaceae Andrographis genus that widely grows in Fujian, Guangdong, Guangxi and other provinces in China, has pharmaceutical effects of heat clearance, detoxication, blood cooling and detumescence [1]. Its extracts are often used as raw materials to prepare andrographis injections [2]. Diterpene lactones of small molecular compounds, including andrographolide (AND), neoandrographolide (NAD), and dehydroandrographolide (DDA) (Figure 1), are regarded as the major effective components [3]. Clinically, andrographis injection offers beneficial effects in the treatment of respiratory tract infections, acute bacillary dysentery, gastroenteritis, fever and other diseases [4]. However, anaphylactoid reactions as a result of andrographis injection have recently been repeatedly reported, and these reactions occur when the patient first comes in contact with the andrographis injection [5]. At present, the available methods for evaluating the anaphylactic reaction induced by traditional Chinese medicine injection (TCMI) are mainly used to detect the sensitization of the protein antigen, which appears as a false-negative result regarding a low-molecular-weight compound (LMWC) [6]. Unfortunately, there are still no reliable models for LMWC to examine anaphylactoid reactions.

The popliteal lymph node assay in mice is considered a promising and unique approach for assessing the immuno-sensitization potential of LMWCs and has the potential to be used as a screening tool for immuno-toxicological hazards identification [7]. The mast cell degranulation test is a common *in vitro* method for evaluating the adverse effects of drugs and has been widely used to examine allergenicity and to perform immunotoxic-mechanism research [8]. The potential sensitizing constituents of an andrographis injection have not been elucidated. Therefore, in our study, the direct popliteal lymph node assay (D-PLNA) in mice and a model of P815 mast cell degranulation *in vitro* were used to evaluate the anaphylactoid reaction induced by extracts from *Andrographis paniculata* Nees.

## Materials and methods

### Test compounds and other reagents

**Figure 2:**
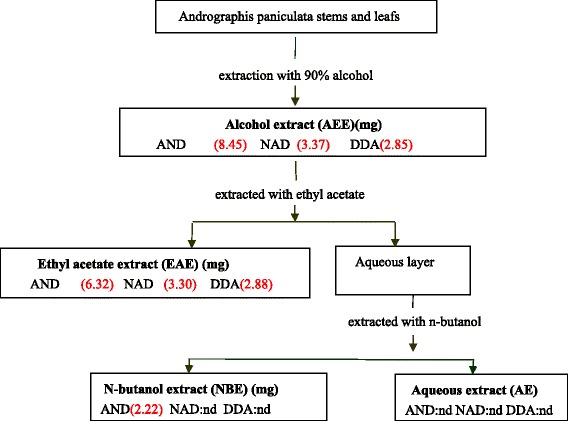
Production and AND, NAD and DDA contents of the extracts and extract fractions from *Andrographis paniculata* stems and leaves. AND: andrographolide, NAD: neoandrographolide and DDA: dehydroandrographolide. nd: not detected. The substance was monitored by TLC and quantified by HPLC.

Andrographis injection (CAS120311) was purchased from the MingXing Pharmaceutical Factory. The following test compounds were provided by Professor Longxiaoying from GuangDong Pharmaceutical University, and the preparation of the extracts and extract fractions is shown in Figure 2. The purity of dehydroandrographolide (DDA), neoandrographolide (NAD) and andrographolide (AND) was greater than 98%. Dimethylsulfoxide (DMSO) (CAS07S00415), C48/40 (CAS040M4098) and MTT (CAS05710250) were purchased from Sigma Chemical (USA). Bovine serum albumin (BAS) and neutral red dye (CAS85R12098) were purchased from Beijing Dingguo Biological Reagents Company (China). Mouse histamine kits (CAS02071110, RD, USA), DMEM medium, RPMI-1640 medium (CASNVH0302), fetal bovine serum (CASNVJ0113) and penicillin-streptomycin (CASJ101651) were purchased from Hyclone, USA. Diclofenac sodium (DF) (CAS15307-79-6) was obtained from GuangZhou Feibo (China).

### Instruments

A TMS-F microscope (Nikon, USA), a Precison5410-220 CO_2_ incubator (Thermo Scientific, USA), a Universal1320R supercentrifuge (Hettitch, GER), an ELIASA-680 instrument (Bio-Rad, USA), 5471R refrigerated centrifuge (Eppendorf Centrifuge, USA), and a CountstarIC1000 instrument (RuiYu BIO-TEC, China) were used in this study.

### Animal

Female Balb/c mice (20 ± 2 g) were obtained from the GuangDong Medical Laboratory Animal Center (China). All of the mice were individually housed in cages containing bedding material, exposed to a 12-h light/12-h dark cycle (lights on at 08:00) and provided food and water *ad libitum*. The studies were performed in accordance with the proposals of the Committee for Research and Ethical Issues of the International Association and were approved by the Committee on the Use of Human and Animal Subjects in Teaching and Research of Guangdong Pharmaceutical University.

### Preparation of extracts and extract fractions

*Andrographis paniculata* stems and leafs were extracted three times with 90% alcohol (6.7 L each) at 60°C for 12 h per extraction. The mixture was filtered through gauze and then evaporated with a rotary evaporator (RE-52A, Shanghai Rongya Labortechnik, China) to 1/13 of the initial volume. The alcohol concentration of the extract was adjusted to 45% with 95% alcohol, and 3% ~ 4% active carbon (m/v) was added to the backflow for 30 min. The mixture was filtered and evaporated to remove the ethanol and thus obtain the alcohol extract (AEE). The AEE was dissolved in pure water at 60°C and became a uniform suspension liquid. The suspension was extracted by adding ethyl acetate to obtain the ethyl acetate extract (EAE). The aqueous layer was then extracted further by adding n-butanol to obtain the n-butanol extract (NBE). The remaining aqueous phase was the aqueous extract (AE). The production and composition of the extracts and extract fractions are shown in Figure 2.

### Preparation of AND, NAD and DDA

**Figure 3:**
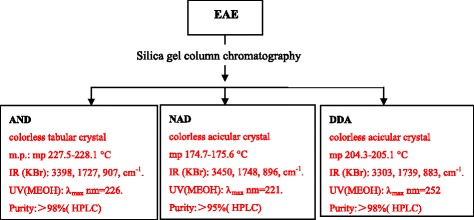
Preparation of AND, NAD and DDA from EAE. AND: andrographolide, NAD: neoandrographolide and DDA: dehydroandrographolide. EAE: ethyl acetate extract. The substances were detected by thin-layer chromatography.

The EAE was separated by means of silica gel column chromatography (column length 90 cm, internal diameter 10 cm) using petroleum ether and ethyl acetate as the eluent. Then, andrographolide (AND), neoandrographolide (NAD) and dehydroandrographolide (DDA) were obtained (TLC monitoring) as shown in Figure 3. The AND, NAD and DDA contents of EAE are.

### HPLC analysis of AND, NAD, and DDA

The identifications of AND, NAD, DDA were performed according to conventional approach for known structure: crystal form (Optical microscope, Shanghai Guangmi instrument Co. Ltd, in P.R. China, XSP-2C), Melting point(Yuhua instrument Co. Ltd. in P.R. China, Micro melting point apparatus X-4), IR ( PerkinElmer, American, Spectrum-100), UV (SHIMADZU,Japan, UV-2450 ) and HPLC (Waters ChemStation, Breeze2, USA).

**Figure 4:**
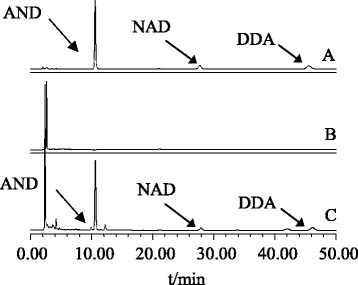
HPLC chromatograms of three diterpenoids in *Andrographis paniculata*. **A**. Three standard diterpenoids; **B**. Blank sample; and **C**. Sample of AEE.

The quantitative study of AND, NAD, and DDA in *Andrographis paniculata* and extracts from its processing by-products was performed by HPLC (Figure 4). The HPLC system (Waters ChemStation, Breeze2, USA) consisted of a low-pressure binary pump (model Waters 1525), a UV detector (model Waters 2489) and an autosampler (model Waters 2700 Autosampler) with a 48-vial capacity sample. The separations were carried out on a Phenomenex RP-18 column with a particle size of 250 mm (5 μm). A Phenomenex RP select B guard column with a particle size of 5 μm was placed in front of the analytical column. The chromatographic conditions were as follows: filtered (22 μm) acetonitrile and water, gradient programmed isocratic elution, room temperature, run time of 50 min, injection volume of 10 μl, and wavelength of 225 nm.

### Evaluation of the anaphylactoid potential using a direct popliteal lymph node assay (D-PLNA) in mice

#### Analysis of the effects of andrographis injection through a popliteal lymph node assay in mice

The assay was performed according to the published methods [9,10]. Sixty female BALB/c mice were randomly divided into six groups: NS group, control group, 3% Tween 80 group, andrographis injection group, 20% DMSO group and diclofenac sodium (DF) group. On day 1, the mice in the control group received a single subcutaneous injection of saline (50 μl) with a volume of 1 ml in the heel-toe direction on the right hind footpads using a microsyringe. Then, 3% Tween 80, andrographis injection, 20% DMSO and DF (50 μl) were administered to the animals belonging to the corresponding groups in the same manner. The NS group mice did not receive any treatment. The test substances were dissolved in saline to prepare the appropriate solutions. All of the solutions with the exception of DMSO were filtered through a 0.2-μm microporous membrane prior to injection. On day 7, the mice were sacrificed by cervical dislocation, and the PLNs from both the right and left sides were removed in an aseptic operation room, soaked in 75% ethanol for 10 min and placed in ice-cold 1% BAS-PBS. After removing the excess fatty tissue, the weight of the PLNs was measured. The weight index was defined as WI = the lymph node weight of right side/the lymph node weight of left side.

The PLNs were then fixed in a paraformaldehyde solution, embedded with paraffin, sliced and stained with HE. The histopathological changes of the PLNs were observed under an optical microscope.

### Evaluation of the anaphylactoid potential of the different extracts and extract fractions from *Andrographis paniculata*

Fifty female BABL/c mice were randomly divided into five groups: DF group, AE group, EAE group, NBE group and AEE group. The test substances at a dosage of 2 mg in 50 μl were dissolved with 20% DMSO solution and then injected into the right hind foot; the left hind foot was injected with same volume of vehicle. This method was based on published protocols [9,10]. On day 7, the mice were sacrificed, and the weight (WI) and cellularity (CI) indices were determined.

Preparation of cell suspensions: After removing the excess fatty tissue and weighing the samples, the PLNs were quickly placed in ice-cold 1% BAS-PBS, and the lymph nodes were then placed on a 200-mesh stainless steel mesh and ground lightly with the inner core of a syringe while washing with 1% BAS-PBS into a small Petri dish. The cell suspension was collected and centrifuged at 2200 r/min for 5 min, the supernatant was discarded, and 0.5 ml of ice-cold 1% BAS-PBS was added while resuspending the cells several times. An automatic cellular counter was used to count and record the number of cells surrounding the lymph nodes and to obtain photographs. CI = the lymph node cell number of right side/the lymph node cell number of left side. WI > 2 and CI > 5 were considered a positive response, suggesting that the test substance has sensitization potential.

### Evaluation of the anaphylactoid potential of the monomers of andrographis

To study the potential sensitization of three monomers of andrographis (NAD, DDA and AND), D-PLNA was performed using NAD, DDA and AND. Thirty female BABL/c mice were randomly divided into three groups as follows: AND group, DDA group and NAD group. The test substances at a dosage of 1 mg/50 μl were dissolved in a 20% DMSO solution and then injected into each mouse. This method is based on published protocols [9,10].

### Evaluation of the anaphylactoid potential using the P815 mast cell degranulation model *in vitro*

#### Cell culture

P815, a mast cell line from mouse tumor cells, was provided by FuNing, PhD, Department of Immunology, Southern Medical University. The cells were incubated with DMEM medium containing inactivated 10% fetal bovine serum and penicillin-streptomycin (0.6 × 10^5^ u · L^−1^) at 37°C and 5% CO_2_ saturated humidity. The test was performed when the cells were in the logarithmic phase of growth.

### Determination of P815 mast cell degranulation and the release of bioactive mediators

After 48 h, the cells in each well were washed three times and balanced for 10 min with 1 ml of a Tyrode solution at 37°C and 5% CO_2_. The test groups received 50 μl of the optimal concentration of the extracts and extract fractions of andrographis (20.42 ng/ml AEE, 16.98 ng/ml EAE, 14.66 ng/ml NBE, 7.00 ng/ml AND, 9.48 ng/ml DDA, and 0.48 ng/ml NAD), whereas the vehicle group only received 50 μl of the Tyrode solution. The positive group was challenged with 50 μl of C48/80 (10 μg/ml). After 45 min, the responses were stopped. The cell supernatants and cells were collected by centrifugation at 2000 r/min and 4°C for 10 min) to determine the bioactive mediators.

#### Measurement of histamine levels

The histamine levels in the supernatant were determined and analyzed using a mouse histamine ELISA kit as described by the manufacturer.

#### Measurement of ammonia glycosidase release

A 100-μl aliquot of the supernatant was added into a 96-well plate and was later mixed with 1 ml of amino hexose solution. The plate was maintained at 37°C with 5% CO_2_ for 45 min. The reaction was then terminated with 150 μl of termination liquid (0.1 mmol/L Na_2_CO_3_/NaHCO_3_ buffer solution). The OD value of the reaction liquid in each well was then determined at 405 nm. The release ratio of ammonia glycosidase was calculated by dividing the amount of ammonia glycosidase released by the test group by that released by the positive group after deducting the amount released by the vehicle group.

#### Measurement of cell degranulation levels

The cells were washed twice with PBS and then added to a 24-well plate with 0.3% neutral red dyeing liquid (500 μl/well). The profile of the culture plate was then gently tapped. After dyeing for 3 min at 37°C, the number of degranulated cells in a set of 100 randomly selected cells was counted. The cell degranulation percentage was calculated by dividing the number of degranulated cells by the total number of cells.

### Statistical analysis

The data were analyzed using the SPSS.17.0 software. SNK test was used to compare two populations. One-way ANOVA followed by Fisher’s least significant difference (LSD) test for pairwise comparisons was used for comparisons of more than two populations. P < 0.05 was considered significant.

## Results

### Analysis of the effects of andrographis (CXL) injection through the popliteal lymph node assay in mice

**Figure 5:**
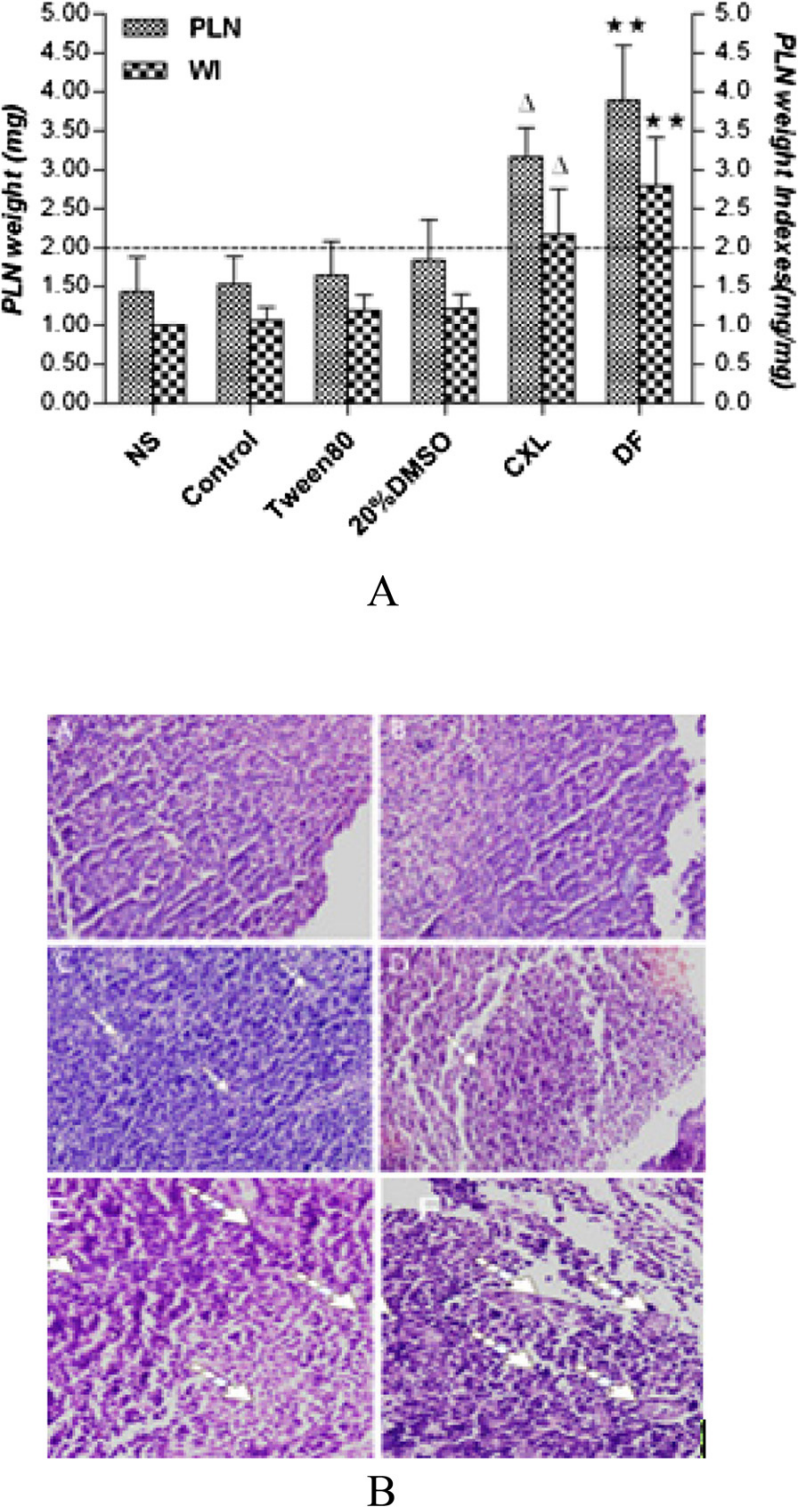
Effects of andrographis injection on PLN weight in BALB/C mice. (**a**) WI and PLN weight of the treated groups. The measured WI and PLN weight show significant increases in the andrographis injection group compared with the 3% Tween 80 group, and no significant differences were observed between the 20% DMSO and 3% Tween 80 groups relative to the control group (saline group). The data are presented as the means ± SD from 10 mice per group. ^△^P < 0.05 vs. the 3% Tween 80 group, ^★★^P < 0.01 vs. the control group. (**b**) Histopathological findings in the PLNs. (**A**) Normal group, (**B**) Control group, (**C**) 3% Tween 80 group, (**D**) 20% DMSO group, (**E**) andrographis injection group, (**F**) DF group. H&E stain; magnification, 400×.

As shown in Figure 5(a), 3% Tween 80 and 20% DMSO caused a slight increase in the popliteal lymph node weight compared with the normal saline group (control group), but the WI was lower than 2, suggesting that 3% Tween 80 and 20% DMSO have no potential allergenicity. Compared with 20% DMSO, andrographis injection and DF induced a significant increase in the PLN weight with WI > 2, suggesting that andrographis injection exhibits potential allergenicity.

The observed popliteal lymph node pathology showed that the structure of the PLNs in the normal group (Figure A) and the control group (Figure B) constituted mature lymphocytes with cortical and clear lines in the subcortical areas and a small number of lymphoid follicles. The structure of the PLNs in the 3% Tween 80 group was normal, with mostly small, mature lymphocytes within a small amount of tissue (Figure C). The structure of the PLNs in the 20% DMSO group involved smaller secondary cortical areas of high endothelial venules (HEVs) (Figure D). The andrographis injection group (Figure E) showed varying degrees of cortical and blurry subcortical lesion area boundaries, an increase in the HEV cross-section, the presence of a large number of mature transformed cells and other changes. The characteristics of the DF group included an obvious germinal center and an increase in the HEV cross-section upon epithelium cubing into the blood vessel cavity (Figure F).

### Effects of extracts and extract fractions on PLNA in BALB/c mice

**Figure 6:**
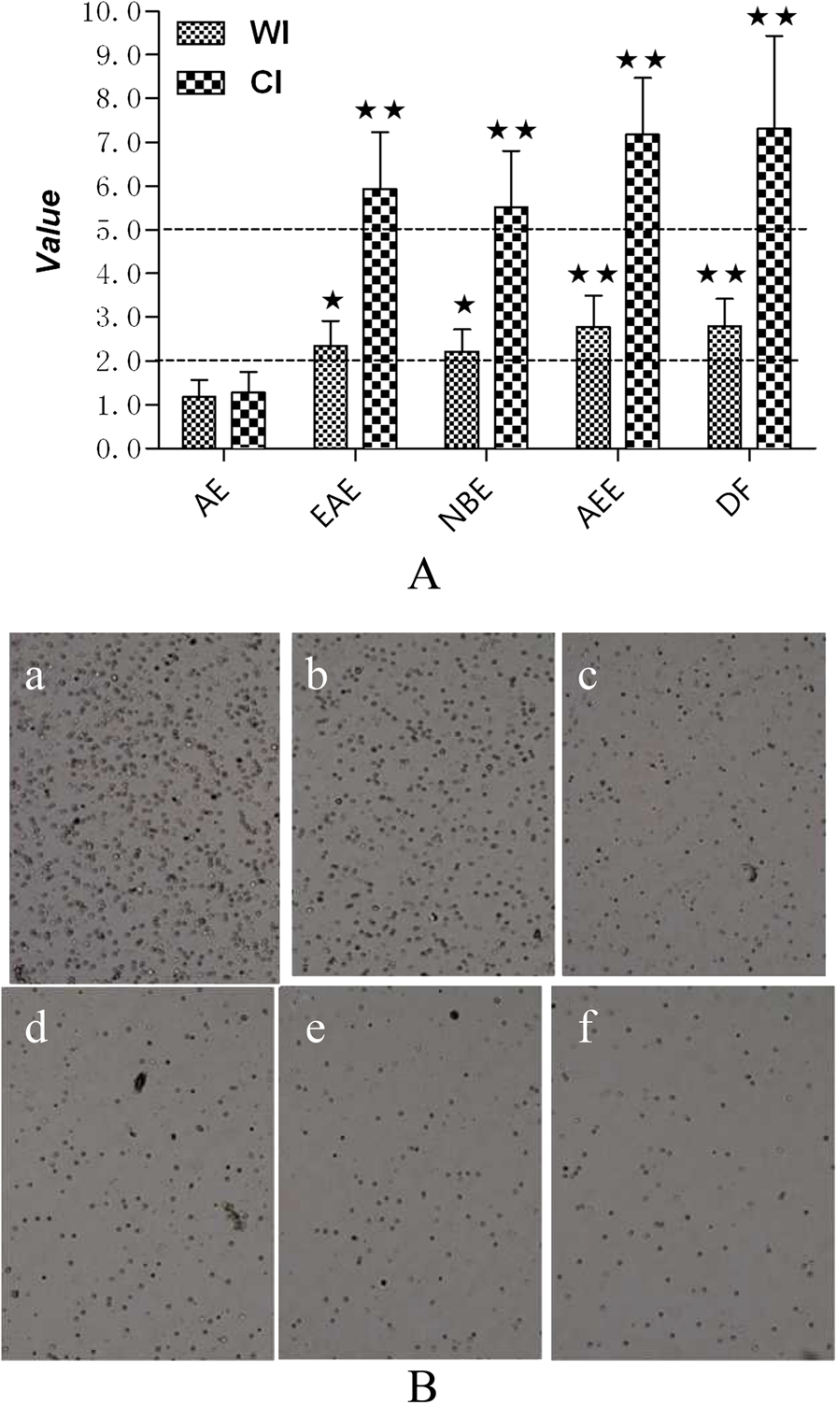
Effects of extracts and extract fractions on PLNs in BALB/c mice. (**A**) WI and CI in the PLNs of treated mice. Compared with the untreated side of each group, the WI and CI values were significantly increased by EAE, NBE and AEE (WI > 2 and CI > 5), whereas AE showed no marked difference. The data are presented as the means ± SD from 10 mice per group. ^★^P < 0.05 and ^★★^P < 0.01 vs. the untreated side. (**B**) Photographs of the PLNs in the cell suspensions from the treated side. DF, AEE, EAE and NBE caused elevations in the number of PLNs. The photographs were taken using a CountstarIC1000 automatic cell counting instrument. **a**, DF group; **b**, AEE group; **c**, EAE group; **d**, NBE group; **e**, AE group; **f**, normal group.

As shown in Figure 6, AEE, EAE, and NBE induced an increase in the PLN weight and cellularity and caused a positive response with WI > 2 and CI > 5, indicating that the extracts and extract fractions of andrographis with the exception of AE have potential sensitivity. Based on the analysis of the components of the different extracts (see diagram), the rest of the extracts with the exception of AE contained diterpenoid lactone constituents, indicating that diterpenoid lactone may be the potential allergen.

### Effects of the andrographis monomers on PLNA

**Figure 7:**
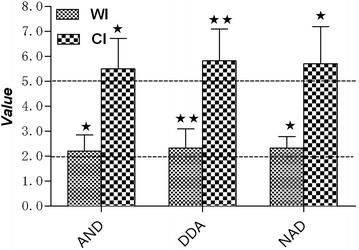
WI and CI in PLNs from mice treated with 1 mg/mice AND, DDA or NAD. The data are presented as the means ± SD from 10 mice per group. ^★^P < 0.05 and ^★★^P < 0.01 vs. the untreated side.

As shown in Figure 7, AND, DDA and NAD induced a positive response with WI >2 and CI > 5, suggesting the potential sensitization of the three monomers of andrographis.

### Effects of extracts and extract fractions and the monomers of andrographis on P815 cell degranulation

**Figure 8:**
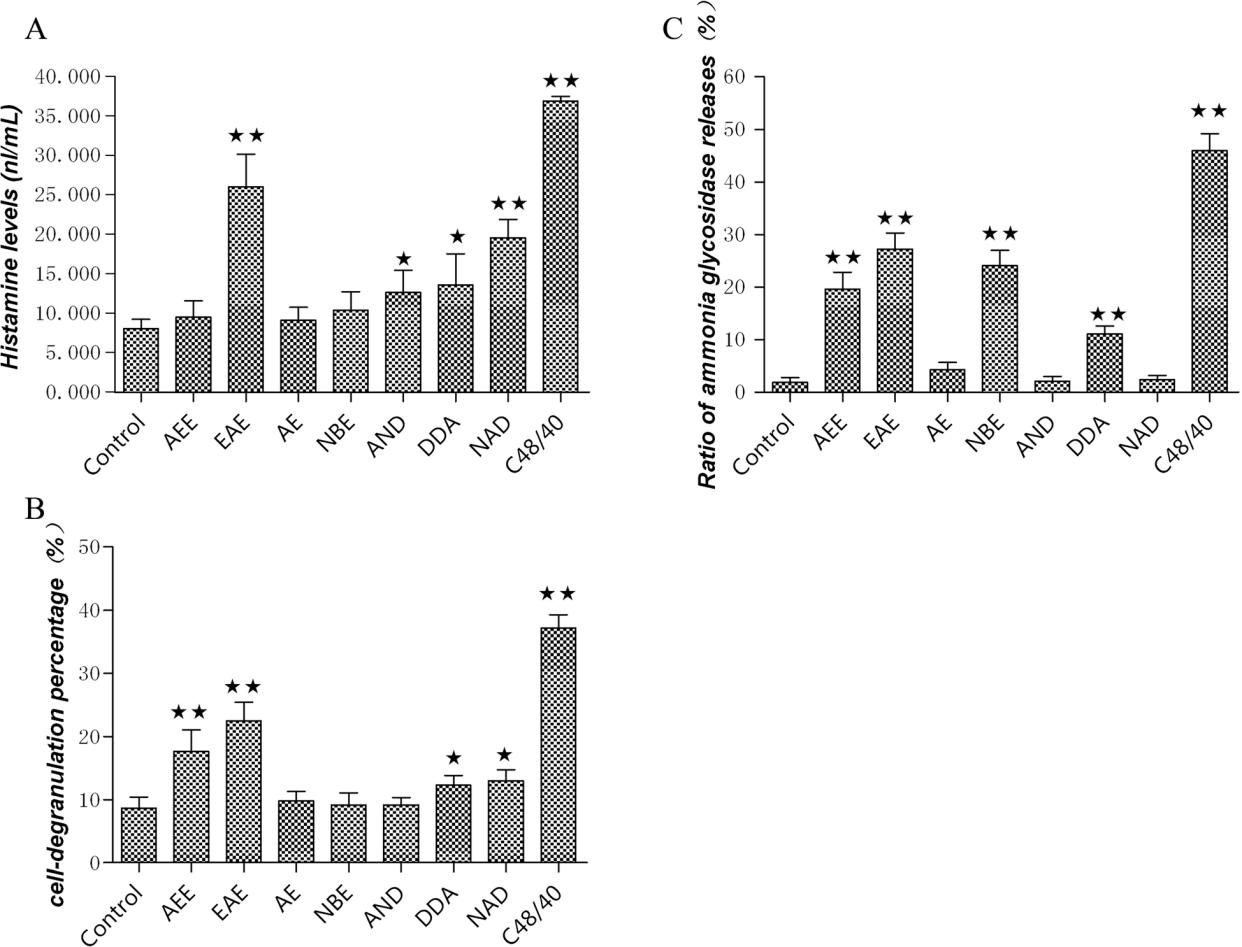
Effects of extracts and extract fractions and AND, DDA, and NAD on P815 cell degranulation. The level of histamine (**A**), the ratio of ammonia glycosidase released (**B**) and the cell degranulation percentage (**C**) were measured. C48/40: the positive group. The data are presented as the means ± SD from three independent experiments. ^★^P < 0.05 and ^★★^P < 0.01 vs. the control group.

To further verify the reliability of the results obtained in the animal experiments, the mast cell degranulation model was applied to these extracts (shown in Figure 8). The results showed that AEE can enhance the rates of ammonia glycosidase release and cell degranulation but has little effect on the histamine content. Among the three different extracts, EAE can improve all three indicators, and NBE significantly improves the ammonia glycosidase release rate, whereas AE had no obvious effect. These results suggest that AEE, EAE and NBE have the potential to exert allergenicity on P815 cells.

The analysis of the three andrographis monomers revealed that AND can increase the histamine content, that NAD can significantly increase the histamine content and degranulation rate, and that DDA has a marked effect on the histamine level, release of ammonia glycosidase and cell degranulation rate, suggesting that the three andrographis monomers have the potential to exert allergenicity on P815 cells.

## Discussion

The popliteal lymph node assay, which is mainly used for the induced phase of drug sensitization and that uses the lymph proliferation of local drainage as the observation end point and the lymph node activation, proliferation, differentiation and secretion of cytokines and antibodies as the observation objective, is currently recognized as the most reliable and efficient method to screen for the allergenicity and immune toxicity of small molecule compounds [7]. Liu Zhaohua applied PLNA to filter the allergenic ingredients of Shuang Huang Lian injection and found that chlorogenic acid itself exerts no sensitization effect [11]. In addition, PLNA has also been successfully used to evaluate the immuno-toxicity of interferon [12]. In addition, researchers have investigated more than 130 compounds that can immune-stimulate the human body and have concluded that PLNA is presently the only reliable method for screening the sensitization potential of small molecule compounds in whole animal experiments [7,13].

Our experimental results show that andrographis injection has allergenicity potential. In addition to the active ingredients, the injection also contains a small amount of excipient Tween 80. However, researchers have confirmed that Tween 80 itself causes irritation, which can result in allergic reaction in animals [14]. The reaction intensity is associated with the dose, concentration and administration rate. Reducing the dose of Tween 80 can decrease or even eliminate the occurrence of allergic reactions [15,16]. Our experimental results show that the 3% Tween 80 group exhibited WI < 2, indicating that the formulated amount of Tween 80 cannot cause an allergic reaction. Therefore, the occurrence of an allergic reaction is likely to be related to andrographis extracts. According to the experimental results, the WIs of the solvents in both the control group and the 20% DMSO (which was used to increase the solubility of the test substances) group were slightly higher than that of the normal group but hardly reached the level of the positive standard (WI > 2), implying that the control solvent and 20% DMSO are also not the allergenic ingredients. These results are consistent with the conclusion reached by Karen Friedrich et al. [17] regarding the effect of the solvent DMSO on D-PLNA; hence, the interference of solvent and DMSO can be excluded. Diclofenac sodium, an antipyretic analgesic, can clinically cause allergic reactions and is thus widely used as a positive drug in PLNA research [18].

Our study of extracts and extract fractions indicates that AEE, EAE and NBE but not AE have potential for sensitization, and based on the analysis of the components of the different extracts, AEE, EAE and NBE all contain diterpenoid lactone constituents, whereas AE does not. Based on the results of our subsequent studies on the three monomers of andrographis, AND, DDA and NAD can cause allergic reactions, implying that diterpenoid lactone has potential for sensitization and that the allergenic ingredients may be AND, DDA and NAD.

The direct effect of drugs or allergens on mast cells and basophil granulocytes, which causes cell degranulation and the release of bioactive mediators, such as histamine and ammonia-glycosidase, is an important approach for the occurrence of an allergic reaction [5,19]. Histamine is a classic iconic material of mast cell degranulation, and ammonia-glycosidase is stored in the secretory granules of mast cells as an active substance; therefore, these bioactive mediators play an important role during the allergic process [20]. Previous studies have shown that ammonia-glycosidase is released in parallel with histamine when mast cell degranulation is activated. For this reason, the ammonia-glycosidase index is also used as a sign of the activation of mast cell degranulation and is determined in combination with histamine [21]. The P815 cell experiments show that the extract fractions with the exception of AE and the three monomers of andrographis are able to induce allergenic reactions and may thus have potential allergenicity. These results are in accordance with the D-PLNA experimental findings and therefore further verify the reliability of the animal experiments.

## Conclusion

In conclusion, our study indicates that AEE, EAE, and NBE as well as the three monomers of andrographis, i.e., NAD, AND and DDA, significantly increase the weight and cell number of PLNs. Additionally, the different extract fractions (AEE, EAE and NBE) and the three monomers of andrographis all markedly elevated the level of histamine, the percentage of cell degranulation and the ratio of ammonia glycosidase released in the cell model. The results of this study suggest that *Andrographis paniculata* extracts have a potential sensitizing capacity and that the allergenic ingredients may be the diterpene lactone compounds of *Andrographis paniculata* Nees.

## Abbreviations

D-PLNA: Direct popliteal lymph node assay
PLN: Popliteal lymph node
WI: Weight index
CI: Cellularity index
AEE: Alcohol extract
EAE: Ethyl acetate extract
NBE: n-butanol extract
AE: Aqueous extract
AND: Andrographolide
NAD: Neoandrographolide
DDA: Dehydroandrographolide
TCMI: Traditional Chinese medicine injection
LMWC: Low-molecular-weight compound
DF: Diclofenac sodium
HEV: High endothelial venules
